# Risk factors for scrub typhus infection in South India: population-based cohort study

**DOI:** 10.1017/S0950268825100484

**Published:** 2025-08-26

**Authors:** Wolf-Peter Schmidt, Neal Alexander, Winsley Rose, Daniel Chandramohan, Mary Cameron, Kundavaram Abhilash, Punam Mangtani, Carol Devamani

**Affiliations:** 1 Department of Disease Control, London School of Hygiene and Tropical Medicine, London, UK; 2 MRC International Statistics and Epidemiology Group, London School of Hygiene and Tropical Medicine, London, UK; 3 Department of Child Health 3, Christian Medical College, Vellore, India; 4 Department of Emergency Medicine, Christian Medical College, Vellore, India; 5 Department of Infectious Disease Epidemiology, London School of Hygiene and Tropical Medicine, London, UK

**Keywords:** scrub typhus, zoonotic infection, vector-borne infection, rickettsiaa, epidemiology

## Abstract

Scrub typhus is a mite-borne infection, largely affecting rural populations in many parts of Asia. This cohort study explored socio-demographic, behavioural, and spatial risk factors at different levels of endemicity. 2206 rural residents from 37 villages in Tamil Nadu, South India, underwent a questionnaire survey and blood sampling at baseline and annually over 2 years to detect sero-conversion. Satellite images were used for visual land use classification. Local sero-prevalence was estimated using 5602 baseline blood samples.

Two hundred and seventy cases of seroconversions occurred during 3629 person-years (incidence rate 78/1000, 95%CI 67, 91). Older age was associated with scrub typhus in crude but not in multivariable analysis adjusting for socio-economic factors. By contrast, the increased risk in females compared to males (RR 1.4) was unaffected by adjusting for confounders. In multivariable analysis, agricultural and related outdoor activities were only weakly associated with scrub typhus. However, agricultural activities were strongly associated with scrub typhus if local sero-prevalence was low, but not if it was high. Females were at a higher risk than males in high-prevalence areas but not in low-prevalence areas. To conclude, agricultural activities were not strongly associated with scrub typhus. Transmission within human settlements may predominate in highly endemic settings.

## Key results

- Scrub typhus is a mite-borne bacterial infection emerging as one of the most important causes of severe undifferentiated febrile illness in Asia, especially the Indian Subcontinent.
- Populations involved in agricultural work were previously thought to be at high risk of infection, acquired via contact with infected mite larvae (chiggers) in fields and forest edges.
- Contrary to this view, this study suggests that the bulk of scrub typhus infection may occur within human settlements and that preventive measures may need to address the environment around places of residence.

## Introduction

Scrub typhus is caused by intracellular bacteria of the genus *Orientia* (Family: *Rickettsiaceae*, order: Rickettsiales) [1]. Scrub typhus presents as an acute febrile illness, occurring predominantly in South Asia, East Asia, and Southeast Asia, where the agent is *Orientia tsutsugamushi* [2]. Cases are also reported from Chile [3], caused by *Candidatus* Orientia chiloensis, and the Middle East and East Africa, caused by *Candidatus* Orientia chuto [4, 5]. Infection can lead to acute respiratory distress syndrome (ARDS), shock, renal failure, meningo-encephalitis, and many other complications [6]. In endemic regions, scrub typhus accounts for a substantial proportion of severe undifferentiated fever leading to hospital admission [6–11].

*Orientiae* are transmitted by the bite of trombiculid mite larvae (chiggers) with small mammals such as rodents and shrews acting as main hosts [12] and humans as accidental hosts. Groups regarded as at risk include farmers, military personnel, and travellers during outdoor activities [12]. The Indian subcontinent has emerged as a region with a particularly high burden of scrub typhus [6–8, 10, 11, 13]. In India, scrub typhus occurs not only in mountain and hill regions, such as Himachal Pradesh [14] and Meghalaya [15] but also in low-land regions characterised by a high population density and intense agriculture, such as the Punjab [16], Uttar Pradesh [17], and Tamil Nadu [6]. This stands in remarkable contrast to studies in Thailand and Malaysia, where scrub typhus is concentrated in mountainous forest areas [18–20]. Unlike earlier observations across Asia [12], agricultural and other outdoor activities were only weakly associated with scrub typhus sero-positivity in a sero-prevalence study from South India [21]. In a further sero-survey from South India, agricultural activities were strongly associated with sero-positivity for scrub typhus in peri-urban settings (where overall sero-prevalence was low) but not in agricultural villages marked by a high sero-prevalence, suggesting that risk factors for scrub typhus may differ depending on the level of endemicity [22]. In highly endemic areas, infection may occur within human settlements, for example, in gardens or other human habitats suitable for trombiculid mites [21, 23–25].

In the present study, we used sero-incidence data from a large population-based cohort study [9] to identify socio-economic, occupational, and spatial determinants of scrub typhus infection in a rural setting in South India with varying levels of endemicity. We further explored whether the marked increase in scrub typhus risk with age and in females found in the main study [9] can be explained by behavioural as opposed to biological factors.

## Methods

### Study population

Details of the cohort study, aiming at estimating the incidence of scrub typhus at different levels of severity, have been published [9]. Briefly, we enrolled 32279 individuals of all ages living in 37 villages in two districts of Tamil Nadu (Vellore and Ranipet). Villages were enrolled purposively based on known endemicity defined as (1) a IgG sero-prevalence of *O. tsutsugamushi* infection of at least 15% identified in earlier studies (n = 28 villages) [10, 22] or (2) at least two scrub typhus cases from a village were admitted to the study institution (Christian Medical College Vellore) between 2016 and 2019 (n = 9). All residents who expected to reside for at least 6 months in selected villages were eligible for participation in the main cohort. To estimate sero-prevalence at baseline and the incidence of serologically detected infection, one participant aged 10+ years per household present at the time of enrolment was randomly selected and asked to take part in a sub-cohort (sero-cohort). The sub-cohort was chosen for the present risk factor study because the serological detection of infections independent of symptoms does not depend on the willingness of study participants to report fever episodes. Enrolment was conducted from February to July 2020.

### Questionnaire survey

A questionnaire survey was conducted involving all participants of the sub-cohort. The questionnaire was administered by two trained staff to sero-cohort participants throughout the follow-up period in random order. The questionnaire covered socio-demographic, socio-economic, and occupational variables with particular emphasis on agricultural practices and the keeping of animals. The latter included taking cows or goat for grazing in uncultivated land surrounding a village, a common practice especially among the elderly. Caste was classified as general, other backward class/most backward class (OBC/MBC), and scheduled caste/tribe (SC/ST) [26]. The terms OBC/MBC and SC/ST generally describe historically disadvantaged groups eligible for certain social benefits such as reservation for government positions and access to higher education. The category SC/ST overlaps with the term dalit, used to describe groups that were historically considered ‘untouchables’. We asked for farming practices in the 4 weeks preceding the survey to assess seasonal changes in practices, and separately for farming during the rainy season, coinciding with the bulk of scrub typhus transmission.

### Collection of spatial data

The GPS location of the household of each participant was taken upon enrolment. Google satellite images embedded in QGIS (version 3.16.8) were used to map villages. Village boundaries were demarked visually. Within a buffer zone of 200 m around each village’s boundary, we visually classified land use based on satellite images (Supplementary Figure S1). We chose 200 m as the maximum distance from the village boundary (edge) because the manual classification process was not feasible for larger areas. Land use was categorised as agricultural field, coconut plantation, mango plantation, mixed plantation, empty grassland, thorny bush land (mainly covered by invasive *Prosopis juliflora*), water bodies (mostly non-perennial rivers, canals, and ponds), and built areas. Areas within the village boundary were classified as built. Each village was visited to conduct ground truthing of areas that could not be classified using satellite images.

### Laboratory methods

Participants enrolled into the sero-cohort underwent venous blood sampling at enrolment and between March and June of each year, resulting in a maximum of three samples per participant [9]. After collection, blood samples were brought to the study centre on the same day. Serum was separated from blood cells, divided into up to two aliquots and stored at −70 °C until testing. We used enzyme-linked immunosorbent assays (ELISA) to detect IgG antibodies to *O. tsutsugamushi* (Scrub Typhus Detect, InBios International, Inc., Seattle, WA, USA). This ELISA uses Karp, Kato, Gilliam, and TA716 recombinant proteins of the 56-kD outer membrane protein. All assays were performed using an automated ELISA analyser (Euroimmun Analyzer1, Euroimmun, Lübeck, Germany). We applied an optical density (OD) cut-off of ≥1.0 for IgG to suggest past scrub typhus infection. The IgG assay has not been formally evaluated with respect to sensitivity and specificity in the study area [27]. However, the corresponding IgM assay of the same manufacturer has been shown to have a 92% sensitivity and 94% specificity in a study done at the location of the present study [28]. Using the same IgG assay, we [29] and others have previously shown that most scrub typhus cases show a strong IgG response lasting for over 10 months. A recent study in symptomatic scrub typhus cases in India used a receiver operating characteristic (ROC) curve analysis which revealed an optimal OD value cut-off for Inbios IgG ELISA of 0.9 with a sensitivity of 72% and a specificity of 93.5% [30]. In a sensitivity analysis, we explored the robustness of the findings to changing the OD cut-off from 1.0 to 0.9 and 1.2.

### Statistical methods

We used data from study participants with at least two annual blood samples taken at baseline, midline, or endline to explore risk factors for sero-incidence, i.e. the rate at which study participants who were sero-negative at the first sample converted to sero-positivity at the following sampling round. Time periods where the first sample was already sero-positive were excluded. Since participants were observed for a maximum of two seasons, those sero-converting during the first year were effectively censored for the rest of the time under study. Incidence rate ratios were calculated using complementary log–log models [31], with person-time calculated as the time between sampling rounds. Where appropriate, rate ratios were adjusted for age, sex, and other potential confounding factors, in particular those deemed to reflect socio-economic status (years of education, location of water source, and open defecation). A simplified conceptual framework of plausible causal relationships between the main exposure variables (farming, type of crop, type of animal rearing, and spatial variables), potential confounders, and scrub typhus is shown in Supplementary Figure S2. Location of water source and open defection represent potential confounding variables because these may be related to exposure to vegetation but are also markers of socio-economic status. The effect of biological age was adjusted for years of education to control for secular changes in level of education as younger age groups may have benefitted from a better education. Spatial variables were adjusted for age, sex, years of education and daily hours of field work in the rainy season reported by the participant, which may be related to place of residence. Potential confounding factors, excluding variables on the causal pathway or those acting as colliders [32], were added singly and jointly to explore changes in the effect estimate and collinearity [33]. Confidence intervals of incidence estimates were adjusted for within village correlation using robust standard errors. Descriptively, correlation coefficients between agricultural activities and scrub typhus cases by month were calculated with and without allowing for a one-month lag, based on scrub typhus case numbers published previously [9].

We calculated Moran’s I using the *ncf* package in R (version 4.3.1) to explore spatial autocorrelation of sero-incidence and prevalence at varying distances between residential locations in intervals of 20 m. The distance between a participant’s residence within a village to the village boundary (i.e. closeness to unbuilt land) was calculated using ArcMap 10.8.1 (ESRI). Using the overlap analysis tool in QGIS 3.16, we estimated the exposure to different land use environments for each study participant. The percentage of different land use types within 200 m circles around each household was estimated as a continuous variable ranging from 0% to 100%. A radius of 200 m was chosen as this was the distance between the village edge and areas outside within which the land use classification around each village was conducted. The effect of distance to village edge and land use types on scrub typhus incidence was estimated using Poisson regression accounting for spatial autocorrelation (R packages *spdep* and *spmodel*). In the Poisson models, the time of infection was assumed to be at the midpoint between sampling rounds.

We used the proportion of baseline sero-positive individuals within 200 m buffer circles around each household as a measure for local sero-prevalence. For illustration, local sero-prevalence was categorised as <25%, 25 to <50% and ≥ 50%. Interaction between potential risk factors and local sero-prevalence was explored on a multiplicative scale using Poisson regression accounting for spatial autocorrelation, with local sero-prevalence added as a continuous variable. In addition, we explored interaction on an additive scale (risk differences) by using binomial regression with an identity link, treating sero-conversion after each observation period as a binary outcome. As adjusting for spatial autocorrelation was not possible, we adjusted the additive models for clustering at village level using robust standard errors.

We conducted two sensitivity analyses to better understand the effect of excluding baseline sero-positive participants from the study. First, we expanded the study population of the sero-cohort by including baseline positive participants. We defined serologically apparent infection in this group as a change in the optical density of at least 1.0. In a second sensitivity analysis, we used baseline sero-prevalence instead of sero-incidence as the outcome. In this analysis, we used an optical density of 2.0 instead of 1.0, as the cut-off under the assumption that a higher cut-off may better reflect recent infection. No imputation was done for missing values.

### Ethics

Written consent (or assent in minors) was obtained prior to obtaining a blood sample. The study was approved by Christian Medical College’s Institutional Review Board (Ref: 11726) and London School of Hygiene and Tropical Medicine’s Research Ethics Committee (Ref: 16573). The study is registered at clinicaltrials.gov (NCT04506944) [34]. The study was supported by the Medical Research Council, UK (Grant Ref: MR/S023275/1).

## Results

**Figure 1.**
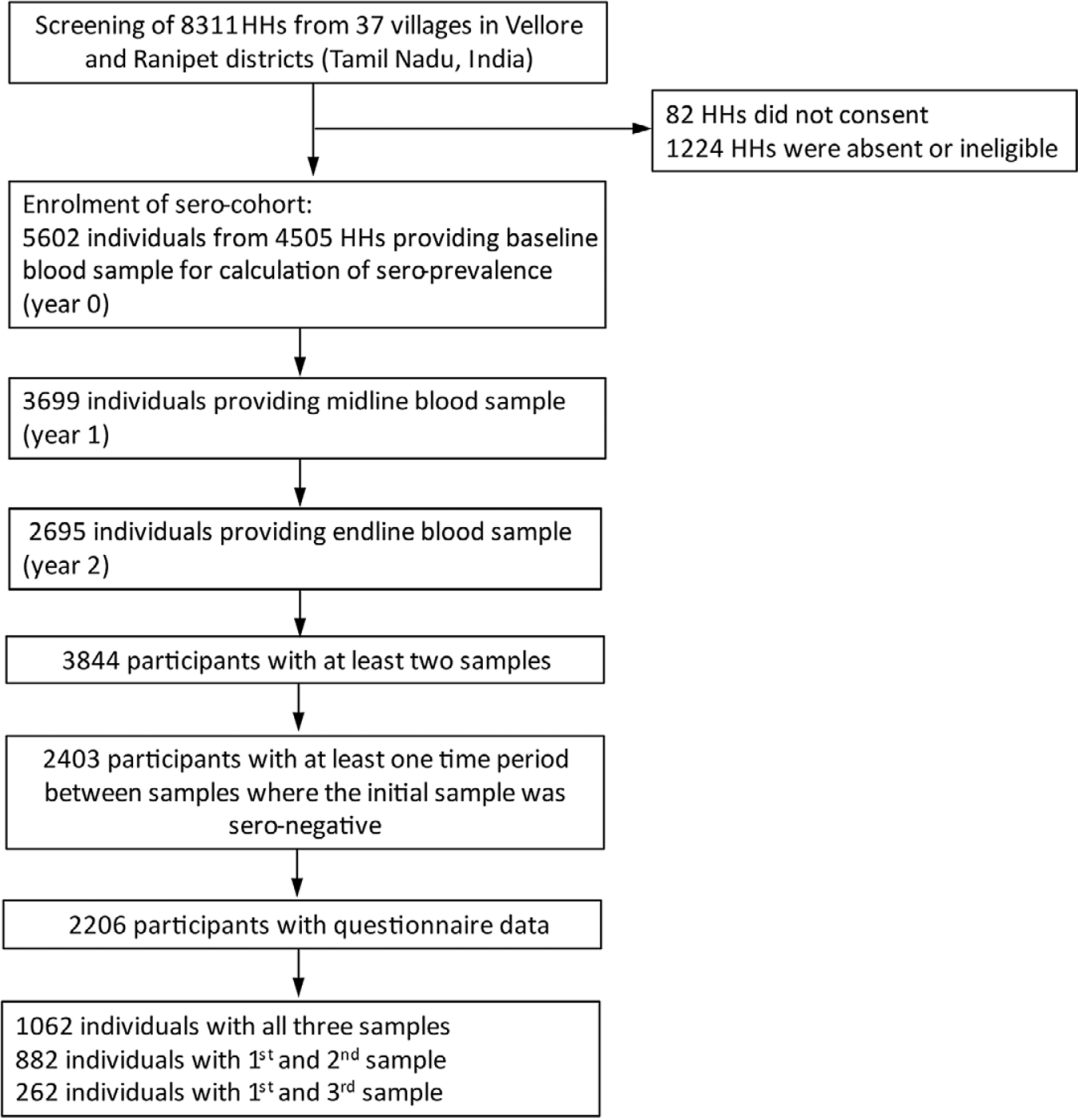
Study flow diagram.

Of 32279 participants in the original study, a baseline blood sample was collected in 5602 for estimating sero-prevalence. At least two annual blood samples were available in 3998 participants, of which 2403 had at least one initial sample which was sero-negative, i.e. they were sero-negative either at baseline or after the first year or both. Of these 2403, questionnaire data were collected from 2206 (92%) (Figure 1). Those without questionnaire data were more often male compared to those with data (44.7 vs. 39.6%, Table 1) and slightly older (38.9 vs. 37.1, Table 1). Among the 2206 participants, 1144 were observed for one time period and 1062 for two periods. Participants observed for two time periods were more often female and were more often involved in full time farming and grazing of animals compared to those observed for only one time period or those who only provided a baseline sample (Supplementary Table S1). The overall sero-prevalence in the baseline sample (n = 5602, Figure 1) was 42.6% (95% CI 35.5%, 50.1%). The overall sero-incidence among the 2206 sero-cohort participants was 78.3 per 1000 person-years (95%CI 67.4, 90.8). Sixty per cent of sero-cohort participants were female (Table 1), compared to only 51.9% females in the main cohort of 32279 participants. The mean age was 37.1 (Table 1) compared to 38.7 in the main cohort (excluding those under the age of 12). Males had a higher mean age, and on average, one year more formal education than females. Most respondents (56%) belonged to the MBC/OBC caste category. Only about 15% of households had their main water source outside the compound. Open defecation was more common in males than females. Comorbidities were equally distributed between sexes. Although farming was common (55% overall), full-time compared to part-time farming was more common in females than males. Females spent more hours per day with farming activities (mean of 2.1 h per day on either agriculture or animal husbandry vs. 1.5 for males). Collecting firewood or vegetation used as animal fodder was slightly more common among females than males (53 vs. 48%).

**Table 1.** Socio-demographic, clinical, and occupational characteristics by sex

	Males	Females	Total	P value^a^
	(*n* = 874)	(*n* = 1332)	(*n* = 2206)	
Age, mean (SD)	38.8 (18.0)	36.1 (35.3)	37.1 (16.1)	<0.001
Age category				<0.001
<20y	17.8	13.4	15.1	
20–29	18.7	22.3	20.9	
30–39	15.9	26.5	22.3	
40–49	17.1	17.7	17.4	
50–59	13.7	11.7	12.5	
60–69	11.9	6.8	8.8	
≥70	5.0	1.7	3.0	
Sex, %	39.6	60.4	-	-
Years of education, mean (SD)	8.7 (4.5)	7.8 (4.4)	8.1 (4.5)	<0.001
Caste, %				0.794
Scheduled caste/tribe	38.9	37.9	38.3	
Most backward class/ Other backward class	55.8	56.3	56.1	
General	5.3	5.9	5.6	
Water source location outside, %	13.6	16.2	15.2	0.096
Open defecation, %	57.6	44.5	49.7	<0.001
Hypertension, %	9.2	8.9	9.0	0.865
Diabetes, %	10.3	9.7	9.9	0.642
Farming, %				<0.001
No	45.2	45.1	45.2	
Part-time	29.8	15.8	21.3	
Full-time	25.1	39.1	33.5	
Farming-hours per day, mean (SD)	1.5 (2.5)	2.1 (3.1)	1.8 (2.9)	<0.001
Taking animals for grazing – hours per day, mean (SD)	0.7 (1.2)	0.6 (1.0)	0.6 (1.1)	0.005
Collecting wood or animal fodder, %	48.1	53.4	51.3	0.016

a males vs. females.

### Socio-demographic and socio-economic risk factors

Sero-incidence strongly increased with age (Table 2), but this association was reduced considerably in multivariable analysis. By contrast, the increased incidence in females compared to males remained after adjusting for potential confounders (rate ratio 1.4). The decrease in incidence for each additional year of schooling was slightly reduced in multivariable analysis. There was no evidence that belonging to a lower caste increased incidence. Having a water source outside the compound (rate ratio 1.2) and leaving the house for open defaecation (rate ratio 1.3) was associated with a higher incidence, but adjusting for confounders substantially decreased these associations. After controlling for age and sex, diabetes, and hypertension were not associated with scrub typhus incidence.

**Table 2.** Sociodemographic and clinical risk factors for sero-conversion

	N	Rate (per 1000 PY)	Crude IRR^a^	95%CI	Adjusted IRR^a^^,^^b^	95%CI
***Sociodemographic factors***						
Age (per 10-year increase)	2206	–	1.10	1.02, 1.19	1.04	0.95, 1.13
Sex						
Male	874	62.2	1.0 (ref)	–	1.0 (ref)	
Female	1332	89.0	1.4	1.1, 1.8	1.4	1.1, 1.8
Years of schooling (per increase in 1 year)	2206	–	0.94	0.92,0.97	0.96	0.93, 1.00
Caste						
Scheduled caste/tribe	834	76.3	1.0 (ref)	–	1.0 (ref)	–
Other backward	1222	78.8	1.0	0.8, 1.3	1.1	0.8, 1.4
General	123	70.8	0.9	0.5, 1.6	1.0	0.5, 1.7
Water source location						
Inside house/compound	1871	75.6	1.0 (ref)			
Outside	335	88.0	1.2	0.8, 1.6	1.0	0.7, 1.4
Open defecation						
No	1106	68.9	1.0 (ref)			
Yes	1094	86.9	1.3	1.0, 1.6	1.1	0.9, 1.5
***Comorbidities***						
Diabetes						
No	1986	75.7	1.0 (ref)		1.0 (ref)	
Yes	219	95.7	1.3	0.9, 1.8	1.0	0.7, 1.5
Hypertension						
No	2006	76.3	1.0		1.0 (ref)	
Yes	199	90.8	1.2	0.8, 1.8	0.9	0.6, 1.4

a IRR – incidence rate ratio (complementary log–log regression).

b All models included age, sex, years of education, daily hours of field work in the rainy season, location of water source and open defecation, except models for comorbidities which were only adjusted for sex and age.

### Occupational and behavioural risk factors

**Figure 2.**
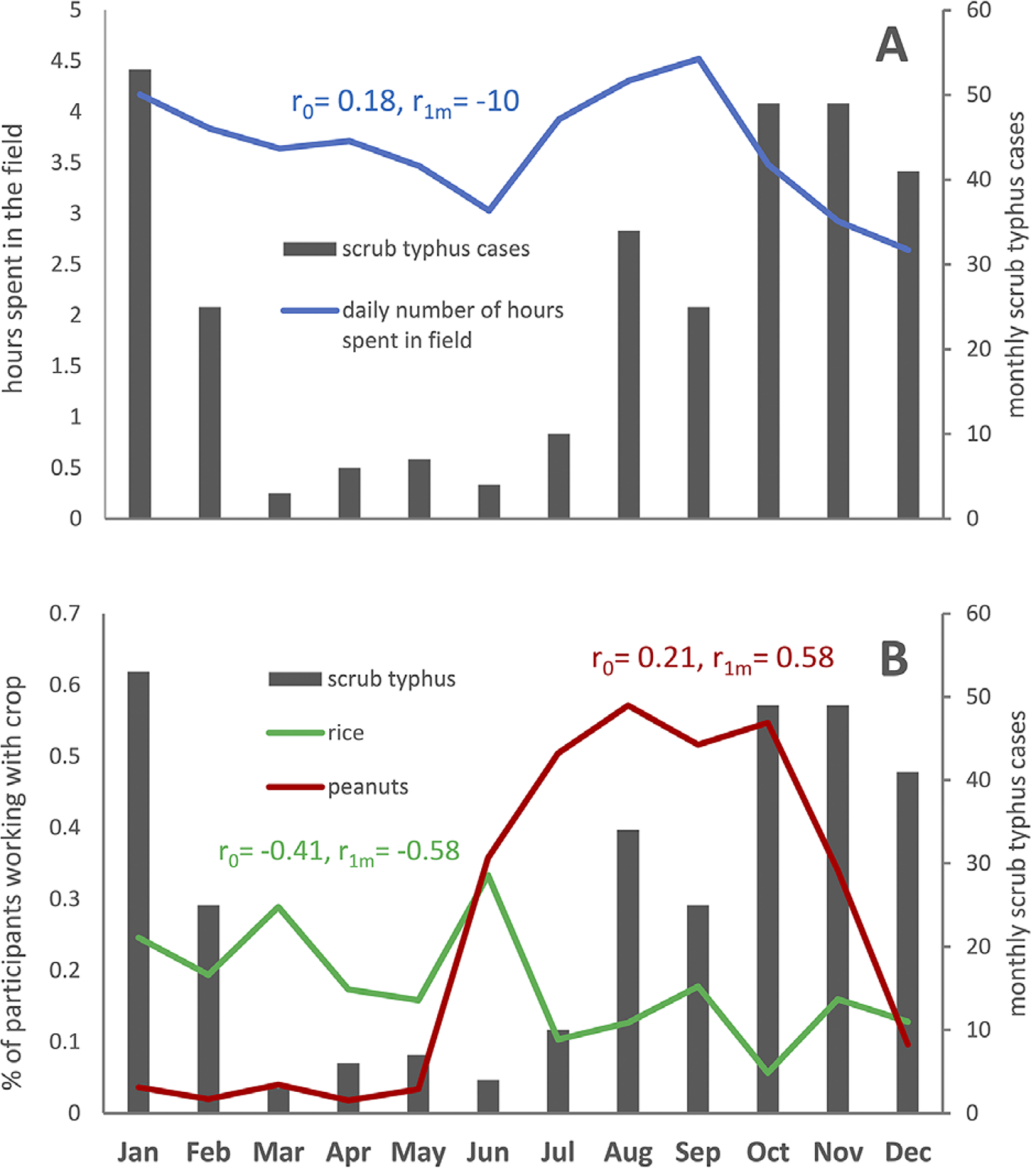
Temporal correlation between farming and scrub typhus: (a) Correlation between the number of daily hours spent with agricultural activities in the field and monthly scrub typhus cases (all study participants included). (b) Correlation between the proportions of participants involved in rice and peanut farming and monthly scrub typhus cases (restricted to participants involved in farming activities]. r_0_ denotes correlation coefficient without lag, r_1m_ denotes correlation coefficient allowing for 1 month lag between activities and scrub typhus cases.

As shown in Figure 2A, there was no correlation between the average number of daily hours spent in the fields by month and monthly scrub typhus cases (r = 0.18), even when allowing for a 1-month lag (r = −0.1). There was a negative correlation between the monthly proportion of participants involved in rice farming and scrub typhus (r = −0.41), in particular when allowing for a one-month lag (r = −0.58). The monthly proportion of participants involved in peanut farming however showed a positive correlation with scrub typhus, when allowing for a 1-month lag time (r = 0.58).

There was little evidence of an association of full-time farming and sero-conversion in multivariable analysis adjusting for confounding (Table 3), nor between the daily number of hours spent in the field during the rainy season (either for agriculture or animal husbandry) and sero-conversion. In the crude analysis, increased risks for sero-conversion were observed for growing peanuts, finger millet, coconut, and mango in the rainy season, which again were substantially reduced in multivariable analysis. Crops that require extensive irrigation, i.e. rice, banana, and sugar cane were not associated with sero-conversion (all point estimates were below 1.0 but the confidence intervals were wide and crossed one). The increased rate of sero-conversion associated with keeping goats was, however, robust to controlling for confounders (Table 3). There was a trend in crude and adjusted analyses for keeping dogs and chicken being associated with sero-conversion, but confidence intervals crossed 1.0. The keeping of cows was not associated with scrub typhus in crude or adjusted analysis. Taking animals for grazing, and collecting grass and firewood were not associated with scrub typhus after controlling for potential confounders.

**Table 3.** Agricultural risk factors for sero-conversion

	N	Rate (per 1000 PY)	Crude IRR	95%CI	Adjusted IRR^a^^,^^b^	95%CI
***Farming***						
None	996	68.3	1.0 (ref)	–	1.0 (ref)	
Part-time	470	70.3	1.0	0.7, 1.4	1.1	0.8, 1.6
Full time	740	94.4	1.4	1.1, 1.8	1.1	0.8, 1.5
Daily hours spent in fields in the rainy season (per 1 h increase)	2206	–	1.04	1.01,1.08	1.02	0.98, 1.05
***Crops cultivated***						
Rice						
No	1907	78.6	1.0 (ref)		1.0 (ref)	
Yes	299	71.0	0.9	0.6, 1.3	0.8	0.6, 1.2
Peanuts						
No	1563	72.1	1.0 (ref)		1.0 (ref)	
Yes	643	90.4	1.3	1.0, 1.6	1.1	0.9, 1.4
Finger millet						
No	2160	76.6	1.0 (ref)		1.0 (ref)	
Yes	46	118.8	1.6	0.8, 3.0	1.5	0.8, 2.9
Coconuts						
No	1971	75.4	1.0 (ref)		1.0 (ref)	
Yes	235	95.3	1.3	0.9, 1.8	1.2	0.8, 1.7
Sugarcane						
No	2171	78.0	1.0 (ref)		1.0 (ref)	
Yes	35	52.2	0.7	0.2, 2.1	0.7	0.2, 2.1
Banana						
No	2147	77.9	1.0 (ref)		1.0 (ref)	
Yes	59	63.3	0.8	0.4, 1.8	0.8	0.4, 1.8
Mango						
No	2093	76.1	1.0 (ref)		1.0 (ref)	
Yes	113	102.1	1.3	0.8, 2.2	1.2	0.8, 2.0
Napier grass						
No	1900	77.7	1.0 (ref)		1.0 (ref)	
Yes	306	76.3	1.0	0.7, 1.4	1.0	0.7, 1.3
***Animal husbandry***						
Cows						
No	1607	80.5	1.0 (ref)		1.0 (ref)	
Yes	599	70.0	0.9	0.7, 1.1	0.8	0.6, 1.1
Goats						
No	2015	74.7	1.0 (ref)		1.0 (ref)	
Yes	191	107.5	1.4	1.0, 2.1	1.3	0.9, 1.9
Chicken						
No	1954	75.1	1.0 (ref)		1.0 (ref)	
Yes	252	95.0	1.3	0.9, 1.8	1.2	0.9, 1.7
Dogs						
No	2029	75.6	1.0 (ref)		1.0 (ref)	
Yes	177	99.1	1.3	0.9, 1.9	1.3	0.8, 1.9
Daily hours spent with grazing animals (per 1 h increase)	2206	–	1.03	0.92, 1.15	1.00	0.88, 1.12
Collecting fodder or wood						
No	1065	72.1	1.0 (ref)		1.0 (ref)	
Yes	1121	83.8	1.2	0.9, 1.5	1.0	0.2, 1.2

a IRR – incidence rate ratio (complementary log–log regression).

b All models included age, sex, years of education and location of water source.

### Spatial risk factors

**Figure 3.**
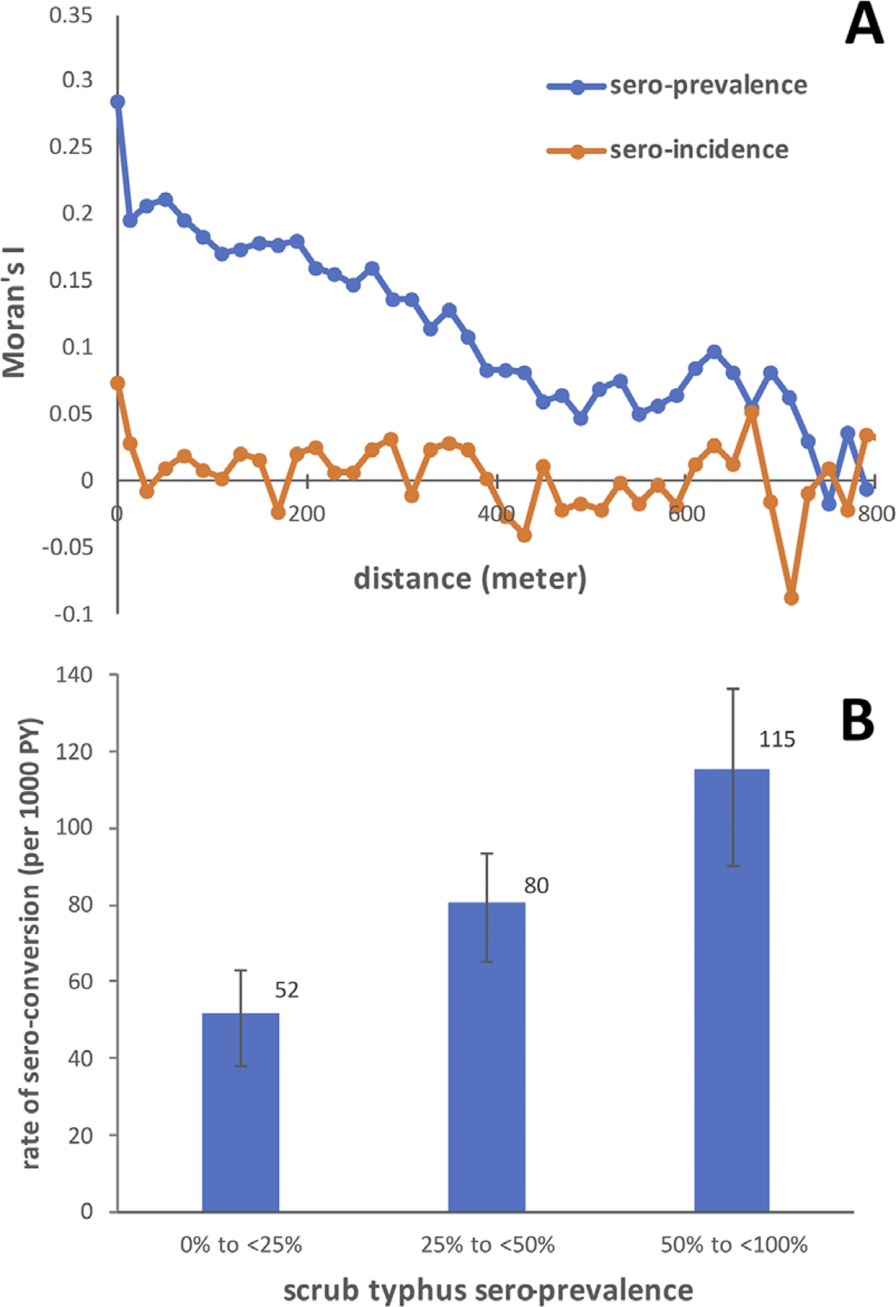
(a) Spatial correlogram displaying *Moran’s I* estimates by distance between household locations of study participants. (b) Association between scrub typhus IgG sero-prevalence within a 200 m radius and sero-incidence.

Sero-prevalence among the baseline sample population showed strong spatial autocorrelation up to a distance of around 750 m (Figure 3A). Except for a distance of zero, there was no evidence for autocorrelation of sero-incidence. Types of land use measured as a proportion within a 200 m radius around a house was not strongly associated with scrub typhus sero-conversion, although there were trends towards an increased risk for tree plantations (mango and mixed plantations), forest, and built area (Table 4). There were trends towards a protective effect associated with empty grassland and thorny bushes. Similarly, water bodies were associated with a reduced risk of sero-conversion. Distance between a house and the edge of the village was not associated with sero-conversion. A higher local scrub typhus sero-prevalence, expressed as sero-prevalence within a 200 m radius of a house strongly increased the risk of sero-conversion (Figure 3B).

**Table 4.** Spatial determinants of sero-conversion

	N	Crude IRR^a^	95%CI	Adjusted IRR^a^^,^^b^	95%CI
**Land use within 200 m of HH (per 10% absolute increase in coverage)**					
Agricultural field	2206	1.03	0.96, 1.1	1.03	0.95, 1.11
Coconut plantation	2206	0.96	0.82, 1.14	0.97	0.83, 1.14
Mango plantation	2206	1.06	0.9, 1.25	1.1	0.96, 1.27
Mixed tree plantation	2206	1.04	0.93, 1.16	1.05	0.95, 1.17
Forest	2206	1.14	0.76, 1.7	1.2	0.78, 1.84
Grassland	2206	0.86	0.66, 1.12	0.92	0.69, 1.22
Thorny bush (mainly*Prosopis juliflora*)	2206	0.91	0.79, 1.05	0.9	0.78, 1.04
Water body (non-perennial)	2206	0.73	0.58, 0.93	0.71	0.56, 0.91
Built area	2206	1.04	0.97, 1.12	1.05	0.98, 1.14
**Distance from house to village edge (per 10 m increase)**	2206	0.98	0.92, 1.03	0.99	0.93, 1.05

a IRR – incidence rate ratio (Poisson regression) adjusted for spatial autocorrelation;

b All models included age, sex, years of education, daily hours of field work in the rainy season and location of water source.

### Risk factors for scrub typhus by local sero-prevalence

**Figure 4.**
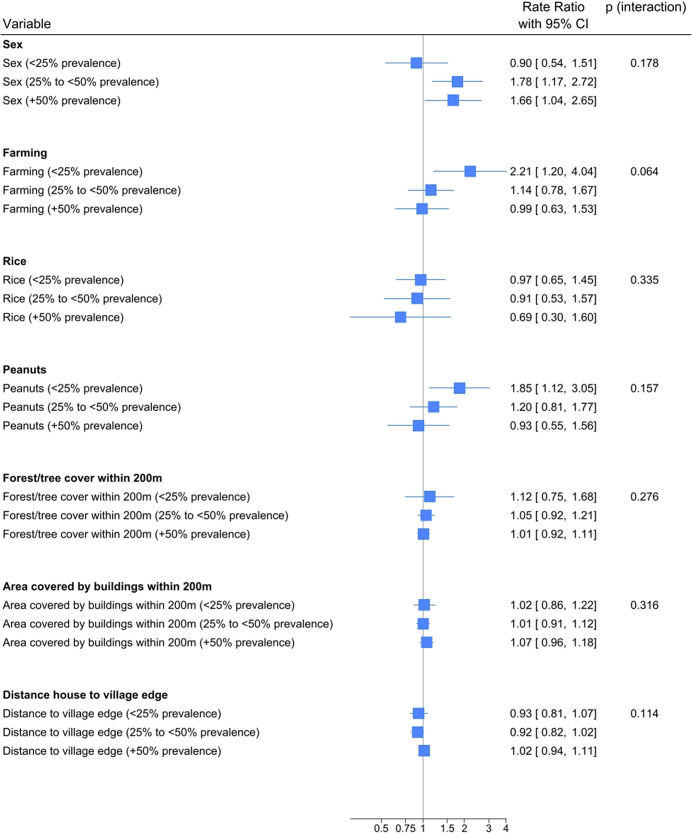
Effect modification between potential risk factors for scrub typhus and local sero-prevalence, categorised as <25%, 25% to <50%, 50% and above, using Poisson regression adjusted for spatial autocorrelation. Tests for interaction were done using sero-prevalence as a continuous variable. Rate ratios are displayed on a log scale.

We explored whether local scrub typhus prevalence modified the adjusted effect sizes of different variables on a multiplicative scale by collapsing the local scrub typhus prevalence within a 200 m radius of a house into three categories (<25%, 25% to <50%, ≥50%, Figure 3B). The effect of sex on the rate of sero-conversion increased substantially with higher local scrub typhus prevalence (Figure 4). By contrast, the effect of farming and growing peanuts on sero-conversion increased with decreasing prevalence. The effect of growing rice on sero-conversion was not strongly modified by sero-prevalence. No strong effect modification was found for distance to the edge of the village and built area, but there was a trend towards proximity to areas of forest/tree plantation (collapsing the variables mango/mixed plantation and forest into one variable – Table 4) being a risk factor at low sero-prevalence but not at high sero-prevalence (Figure 4). We observed similar pattern of effect modification by local sero-prevalence when using risk differences (additive scale) instead of rate ratios (Supplementary Table S2).

### Sensitivity analyses

Using an OD cut-off of 0.9 instead of 1.0 to define sero-conversion slightly reduced the effects of age and sex on the incidence rate (Supplementary Table S3). There were no material changes with respect to farming, daily hours of field work and effect modification of farming and sex by local sero-prevalence. Similarly, increasing the cut-off to 1.2 had little effect on these rate ratios (Supplementary Table S4). Including baseline sero-positive participants in the analysis reduced effect modification of sex by local seroprevalence (Supplementary Tables S5). Using baseline seroprevalence rather than incidence as an outcome attenuated the effect modification of sex and farming by local seroprevalence (Supplementary Table S6). The effect of other exposure variables was largely unchanged.

## Discussion

In this cohort study conducted in a region in South India that is highly endemic for scrub typhus, agricultural activities were only weakly associated with the incidence of serologically detected scrub typhus infection, confirming earlier findings in a similar setting [21]. There was evidence that agriculture was a risk factor for scrub typhus in low prevalence but not in high-prevalence areas. Similarly, the higher incidence of scrub typhus in females observed in this study may depend on local sero-prevalence, with females being at a higher risk than males in high but not in low-prevalence areas.

In highly endemic regions such as the study setting, traditional risk factors for scrub typhus involving agricultural activities appear to lose importance compared to transmission within human settlements [21]. In the study area, women may be more often engaged in domestic activities in the immediate vicinity of their place of residence than men, possibly putting them at risk of infection [24, 25]. Such ‘peri-domestic’ activities may include, for example, washing and drying of clothes and kitchen utensils, cooking outside or tending to pet animals or chicken [25]. The findings are in line with our earlier study that identified agriculture as a strong risk factor for scrub typhus in peri-urban (low sero-prevalence) areas but not in rural villages [22]. Further evidence for most scrub typhus transmission occurring within human settlements may be derived from the spatial analysis, showing that residing closer to the village boundary did not increase risk. In addition, being surrounded by built area within 200 m of a place of residence did not protect from scrub typhus infection but tended to increase the risk (Table 4). The percentage of built area around the place of residence was positively associated with scrub typhus in two out of three study sites from two studies reviewed by Wang and colleagues [35].

The apparent differences in the epidemiology of scrub typhus depending on sero-prevalence may reflect differences in the vector and host biology among settings, and in particular the extent to which trombiculid mites and their hosts have been able to adapt to the peri-domestic habitat. Only few vector studies have directly compared the peri-domestic domain with other habitats. A study from Malaysia found only a low chigger abundance and species diversity in human habitats when compared to forest edges [36]. Conversely, a study from Thailand suggested a higher prevalence of *O. tsutsugamushi* infection in chiggers and small mammals in human settlements and low land agricultural habitats compared to forest [37]. A close association between human habitats and *Leptotrombidium deliense* (an important vector for scrub typhus) has been observed in a study from Yunnan, China, where it was the most commonly found chigger species on small mammals such as rats and shrews caught inside poultry sheds, barns, and residential buildings [38]. In the same study, chigger indices and species diversity were higher in the mountainous regions than in the lowlands, but *Leptotrombidium deliense* was the dominant species in the latter. The type of ecosystem where humans are at greatest risk of scrub typhus infection is likely to depend on chigger density in a given habitat and the amount of time people spend in it. An important factor may be the diurnal behaviour pattern of humans and chiggers, as chiggers have been shown to be most active in the early morning [39], possibly the same time when many peri-domestic activities are performed. Synanthropic small mammals may occur in high densities in human settlements. Even rodents caught in apartment blocks in Lahore, Pakistan have been shown to carry chigger mites [40]. In India, vector studies trapping rodents and shrews in the domestic and peri-domestic environment have demonstrated a high prevalence of *Leptotrombidium* infestation [25, 41, 42], suggesting human settlements as potential areas of scrub typhus transmission, being characterised by a high density of maintaining (small mammals) and accidental hosts (humans). The habitat where small mammals acquire chiggers may, however, differ from the trap location.

The reasons for the strong spatial autocorrelation for sero-prevalence but not sero-incidence (Figure 3A) are unclear but may be due to sero-prevalence unlike sero-incidence reflecting life-time exposure to *O. tsutsugamushi.* The strong association between sero-prevalence and incidence (Figure 3B) may decrease the importance of behavioural risk factors relative to residential location as a risk factor in itself. Nevertheless, our findings confirm studies from Korea suggesting dry farming as opposed to wet farming as a risk factor for scrub typhus [43, 44]. Crops requiring frequent irrigation such as rice, banana, and sugarcane consistently showed rate ratios below 1 (Table 3). Bearing in mind the apparent protective effect conferred by the proximity of non-perennial water bodies (Table 4), it seems that hosts and/or chigger mites avoid land that is subject to periodical flooding, despite the known ability of chigger mites to cope well with flooding [40] and early Japanese studies from the 19^th^ century (reviewed by Chung and colleagues [45]) showing a conspicuous association between flood events and scrub typhus. However, several more recent studies reviewed by Wang and colleagues [35] also found a protective effect of the proximity to water bodies in line with our findings. Associations between scrub typhus and outdoor activities earlier identified as risk factors, such as grass cutting, taking animals for grazing, and open defecation [46], were strongly reduced after controlling for socio-economic confounders (Table 3). Similarly, the increase in the rate of scrub typhus infection with age could largely be explained by agricultural and socio-economic factors, suggesting secular changes such as improvements in education and housing quality among younger generations being protective of scrub typhus.

Residual confounding due to unmeasured or imprecisely measured socio-economic, behavioural and occupational factors is likely to be an important limitation of this study. Behaviours increasing the risk of scrub typhus may involve a large number of peri-domestic and other outdoor activities which are complex and difficult to measure. A more in-depth description of these, for example, by measuring the time different subgroups of the population spend in different environments, especially early in the morning [39] would have strengthened the analysis. Land use classification relied mostly on satellite images, supported by ground truthing. This approach did not account for seasonal changes in land use and water bodies and did not allow classification of different crops within agricultural fields.

The study villages were enrolled on the basis of a known high IgG sero-prevalence of scrub typhus (at least 15%) [34], over-representing highly endemic settings. This may limit the applicability of the findings to other regions with different transmission dynamics. For the analysis of effect modification by local sero-prevalence it would have been informative to enrol more low prevalence villages. Of 2403 individuals eligible for the study, we were unable to obtain questionnaire data in 197 (8%) who were more often male and slightly older. The study sample contained more females than males, as females were more often at home for blood sampling at the time of enrolment and follow-up, a behaviour that, according to our analysis, may also increase their risk for scrub typhus infection. Females were also more likely to participate in all three annual sampling rounds, as were those involved in full time farming and related activities (Supplementary Table S1). The study sample may be biased in other ways such as over-representation of certain occupations allowing them to spend more time at or near their home, for example those involved in animal husbandry or having fields close to their home. Adjusting for age, sex and other confounders is likely to have reduced the influence of selection biases on the effect estimates but may not have fully eliminated it. Children under the age of 10 who had a lower risk of scrub typhus in this study area compared to older ages [9] were not included. Exploring risk factors for scrub typhus in children would have added to this study as children may show different risk behaviours with respect to scrub typhus compared to adults.

By using sero-incidence as outcome, this analysis excluded individuals who were already sero-positive at the beginning of a year under study. High-risk individuals with persistent high antibody titers against *O. tsutsugamushi* may have been under-represented in this analysis. Infection in these individuals may be detected by serial dilution of paired samples, for example, by using immunofluorescence titration assays which we did not do at scale in this study [9]. Using cross-sectional sero-prevalence as an outcome is likely to reflect life-time exposure to *O. tsutsugamushi* [9, 29, 47] and may not allow exploring the effect of current behaviours and place of residence on the risk of infection. Results of sensitivity analyses including baseline sero-positive participants or using sero-prevalence as outcome were broadly compatible with the main analysis (Supplement Tables S5 and S6).

Defining IgG sero-conversion in the absence of universally accepted cut-off points for optical density is a challenge [27], especially with respect to asymptomatic infection. The choice of a suitable cut-off value is made complicated by the dynamic sero-epidemiology of scrub typhus in highly endemic regions where both asymptomatic infection and reversion to sero-negativity are common among permanent residents [9]. Our findings were not materially affected by choosing the lower cut-off of 0.9 identified in a recent study from India [30] or a higher cut-off of 1.2 (Supplementary Tables S3 and S4). However, more work on the definition of serologically confirmed infection independent of symptoms is warranted.

To conclude, agricultural activities and living in close proximity to agricultural fields were not strongly associated with scrub typhus in this highly endemic setting. Our findings suggest that risk factors for scrub typhus may be modified by the level of endemicity, which may explain the contrasting findings from earlier studies [12, 21]. A substantial proportion of scrub typhus transmission in India may occur within human settlements, possibly driving the high burden of scrub typhus in India known from hospital-based studies [11]. Public health measures targeting risk behaviours during agricultural work as have successfully been applied in Japan and Korea [48], may not work in high endemicity settings. However, if most transmission does occur in human settlements, then specific public health measures targeting the life cycle and behaviour of chiggers may be feasible. For example, control of small mammals in villages may reduce the chance of chiggers to mature. Modifications in the built environment may help to reduce human contact with vegetation and soil inside a village [24, 25]. In-depth vector and human behaviour studies are needed to explore the potential of such measures.

## Supporting information

10.1017/S0950268825100484.sm001 Schmidt et al. supplementary material 1 Schmidt et al. supplementary material

10.1017/S0950268825100484.sm002 Schmidt et al. supplementary material 2 Schmidt et al. supplementary material

## Data Availability

Data from this study are available upon request subject to approval by the Institutional Review Board of the Christian Medical College Vellore.

## Supplementary material

The supplementary material for this article can be found at http://doi.org/10.1017/S0950268825100484.
