# Supplementary material for: Risk factors for scrub typhus infection in South India: population-based cohort study
Source: Epidemiol Infect. 2025 Aug 26;153:e102. doi: 10.1017/S0950268825100484 (PMC12455516; doi:10.1017/S0950268825100484)
Supplement: Schmidt et al. supplementary material 2 — Schmidt et al. supplementary material [file S0950268825100484sup002.docx]

***Epidemiology and Infection***

**Supplementary Material:**

**Risk factors for scrub typhus infection in South India: population-based cohort study**

Wolf-Peter Schmidt,^1*^ Neal Alexander,^2^ Winsley Rose,^3^ Daniel Chandramohan,^1^ Mary Cameron,^1^ [Kundavaram PP Abhilash](https://pubmed.ncbi.nlm.nih.gov/?sort=date&term=Abhilash+KP&cauthor_id=25947985),^4^ Punam Mangtani,^5^ Carol Devamani^3^

^1^ Department of Disease Control, London School of Hygiene and Tropical Medicine, London, UK

^2^MRC International Statistics and Epidemiology Group, London School of Hygiene and Tropical Medicine, London, UK

^3^ Department of Child Health 3, Christian Medical College, Vellore, India

^4^Department of Emergency Medicine, Christian Medical College, Vellore, India

^5^Department of Infectious Disease Epidemiology, London School of Hygiene and Tropical Medicine, London, UK

**Table S1**. Comparison of participants with baseline sample only, two samples and three samples

|  | | Baseline sample only | 2 samples | 3 samples | P value |
| --- | --- | --- | --- | --- | --- |
|  |  | (n= 906) | (n= 1144) | (n= 1062) |  |
| Age, mean (SD) | | 36.6 (15.0) | 36.6 (15.9) | 37.7 (16.4) | 0.156^§^ |
| Sex, % | | 53.0 | 56.7 | 64.3 | <0.001 |
| Years of education, mean (SD) | | 8.4 (4.6) | 8.3 (4.4) | 7.9 (4.5) | 0.04^§^ |
| Caste, % | |  |  |  | 0.382 |
|  | Scheduled caste / tribe | 40.5 | 38.3 | 38.3 |  |
|  | Other backward | 55.3 | 56.5 | 55.6 |  |
|  | general | 4.2 | 5.2 | 6.1 |  |
| Water source location outside, % | | 14.4 | 15.0 | 15.4 | 0.820 |
| Open defecation, % | | 46.0 | 51.1 | 48.0 | 0.052 |
| Farming, % | |  |  |  | <0.001 |
|  | No | 56.6 | 46.8 | 43.4 |  |
|  | Part-time | 17.8 | 23.2 | 19.3 |  |
|  | Full-time | 25.6 | 30.1 | 37.3 |  |
| Farming-hours per day, mean (SD) | | 2.1 (3.4) | 2.6 (3.5) | 2.9 (3.6) | <0.001^§^ |
| Taking animals for grazing – hours per day, mean (SD) | | 0.4 (9.4) | 0.5 (1.0) | 0.7 (1.1) | <0.001^§^ |
| Collecting wood or animal fodder, % | | 42.0 | 50.2 | 52.5 | <0.001 |

*chi square test, except ^§^where linear regression was used. Comparison restricted to participants with at least one initial sero-negative sample.

**Table S2.** Effect modification on an additive scale

|  | | N | Adjusted RD^§^ | 95%CI | P (interaction)* |
| --- | --- | --- | --- | --- | --- |
|  | |  |  |  |  |
| **Sex** | |  |  |  | 0.053 |
|  | Prevalence < 25% | 774 | -1.3% | -4.2%, 1.7% |  |
|  | Prevalence ≥ 25%, < 50% | 921 | 2.8% | 0.5%, 5.2% |  |
|  | Prevalence ≥ 50% | 511 | 3.8% | -1.0%, 8.7% |  |
| **Farming** | |  |  |  | 0.111 |
|  | Prevalence < 25% | 774 | 3.5% | 1.3%, 5.7% |  |
|  | Prevalence ≥ 25%, < 50% | 921 | 0.4% | -2.7%, 3.4% |  |
|  | Prevalence ≥ 50% | 511 | 0.0% | -4.4%, 4.5% |  |
| **Rice farming** | |  |  |  | 0.181 |
|  | Prevalence < 25% | 774 | 1.0% | -1.0%, 3.0% |  |
|  | Prevalence ≥ 25%, < 50% | 921 | -1.9% | -5.8%, 1.9% |  |
|  | Prevalence ≥ 50% | 511 | -2.2% | -10.2%, 5.8% |  |
| **Peanut farming** | |  |  |  | 0.481 |
|  | Prevalence < 25% | 774 | 3.6% | 1.0%, 6.2% |  |
|  | Prevalence ≥ 25%, < 50% | 921 | 1.1% | -1.8%, 4.0% |  |
|  | Prevalence ≥ 50% | 511 | 0.0% | -5.0%, 5.2% |  |
| **Forest/tree cover within 200m (per 10% increase)** | |  |  |  | 0.148 |
|  | Prevalence < 25% | 774 | 1.4% | -0.3%, 3.2% |  |
|  | Prevalence ≥ 25%, < 50% | 921 | 0.7% | 0.1%, 1.5% |  |
|  | Prevalence ≥ 50% | 511 | 0.0% | -0.8%, 0.8% |  |
| **Area covered by buildings within 200m (per 10% increase)** | |  |  |  | 0.418 |
|  | Prevalence < 25% | 774 | 0.1% | -0.8%, 1.2% |  |
|  | Prevalence ≥ 25%, < 50% | 921 | -0.3% | -0.9%, 0.3% |  |
|  | Prevalence ≥ 50% | 511 | 0.5% | -0.2%, 1.3% |  |
| **Distance house to to village edge (per 10m increase)** | |  |  |  | 0.482 |
|  | Prevalence < 25% | 774 | -0.3% | -0.9%, 0.3% |  |
|  | Prevalence ≥ 25%, < 50% | 921 | -0.7% | -1.3%, -0.1% |  |
|  | Prevalence ≥ 50% | 511 | 0.1% | -0.8%, 1.2% |  |

^§^Risk difference, using binomial regression with identity link. All models included age, sex, years of education and location of water source. Sex and spatial variables were also adjusted for daily hours of field work in the rainy season. *test for interaction done using likelihood ratio test.

**Table S3.** Sensitivity analysis using a change in ELISA optical density from <0.9 to ≥0.9 define sero-incidence

|  | | | | N | Rate (per 1000 PY) | Adjusted IRR^§^ | 95%CI | P (interaction) |
| --- | --- | --- | --- | --- | --- | --- | --- | --- |
| ***Overall*** | | | | 2168 | 78.2 | - | 67.4, 90.9 | - |
| **Age (per 10 year increase)** | | | | 2168 | - | 1.00 | 0.92, 1.09 | - |
| **Sex** | | | |  |  |  |  | - |
|  | Male | | | 856 | 63.5 | 1.0 (ref) |  | - |
|  | Female | | | 1312 | 86.0 | 1.3 | 1.0, 1.7 | - |
| ***Farming*** | | | |  |  |  |  | - |
|  | | None | | 977 | 69.0 | 1.0 (ref) |  | - |
|  | | Part-time | | 461 | 65.7 | 1.0 | 0.7, 1.4 | - |
|  | | Full time | | 730 | 95.0 | 1.2 | 0.9, 1.6 | - |
| **Daily hours spent in fields (per 1 hour increase)** | | | | 2168 | - | 1.01 | 0.98, 1.05 |  |
| **Distance from house to village edge (per 10m increase)** | | | | 2168 | - | 0.98 | 0.92, 1.04 | - |
| **Effect of sex by sero-prevalence within 200m** | | | |  |  |  |  | 0.0861 |
|  | | | < 25% | 769 |  | 0.8 | 0.8, 1.4 |  |
|  | | | ≥ 25%, < 50% | 903 |  | 1.7 | 1.1, 2.6 |  |
|  | | | ≥ 50% | 496 |  | 1.7 | 1.0, 2.7 |  |
| **Effect of farming by sero-prevalence within 200m** | | | |  |  |  |  | 0.146 |
|  | | | < 25% | 769 |  | 2.1 | 1.2, 3.9 |  |
|  | | | ≥ 25%, < 50% | 903 |  | 1.1 | 0.7, 1.6 |  |
|  | | | ≥ 50% | 496 |  | 1.0 | 0.6, 1.6 |  |

^§^Rate ratio calculated using conditional log-log models (age, sex, farming, daily hours spent in fields in the rainy season), or spatial Poisson models (Distance from house to village edge, effects by sero-prevalence within 200m). All models included age, sex, years of education and location of water source and daily hours of field work in the rainy season, except models with farming as exposure.

**Table S4.** Sensitivity analysis using a change in ELISA optical density from <1.2 to ≥1.2 define sero-incidence

|  | | | | N | Rate (per 1000 PY) | Adjusted IRR^§^* | 95%CI | P (interaction) |
| --- | --- | --- | --- | --- | --- | --- | --- | --- |
| ***Overall*** | | | | 2266 | 75.7 | - | 64.2, 89.1 | - |
| **Age (per 10 year increase)** | | | | 2266 | - | 1.03 | 0.95, 1.13 | - |
| **Sex** | | | |  |  |  |  | - |
|  | Male | | | 896 | 55.4 | 1.0 (ref) |  | - |
|  | Female | | | 1370 | 87.3 | 1.5 | 1.1, 2.0 | - |
| ***Farming*** | | | |  |  |  |  | - |
|  | | None | | 1023 | 64.4 | 1.0 (ref) |  | - |
|  | | Part-time | | 479 | 68.7 | 1.2 | 0.8, 1.7 | - |
|  | | Full time | | 764 | 92.0 | 1.1 | 0.8, 1.5 | - |
| **Daily hours spent in fields (per 1 hour increase)** | | | | 2266 | - | 1.01 | 0.98, 1.05 |  |
| **Distance from house to village edge (per 10m increase)** | | | | 2266 | - | 0.99 | 0.93, 1.05 | - |
| **Effect of sex by sero-prevalence within 200m** | | | |  |  |  |  | 0.207 |
|  | | | < 25% | 784 |  | 1.0 | 0.6, 1.7 |  |
|  | | | ≥ 25%, < 50% | 952 |  | 2.0 | 1.3, 3.0 |  |
|  | | | ≥ 50% | 530 |  | 1.8 | 1.1, 2.9 |  |
| **Effect of farming by sero-prevalence within 200m** | | | |  |  |  |  | 0.017 |
|  | | | < 25% |  |  | 2.4 | 1.3, 4.4 |  |
|  | | | ≥ 25%, < 50% |  |  | 1.1 | 0.8, 1.7 |  |
|  | | | ≥ 50% |  |  | 1.0 | 0.6, 1.5 |  |

^§^Rate ratio calculated using conditional log-log models (age, sex, farming, daily hours spent in fields in the rainy season), or spatial Poisson models (Distance from house to village edge, effects by sero-prevalence within 200m). All models included age, sex, years of education and location of water source and daily hours of field work in the rainy season, except models with farming as exposure.

**Table S5.** Sensitivity analysis on sero-incidence including baseline scrub typhus IgG negative and positive participants

|  | | | | N | Rate (per 1000 PY) | Adjusted IRR^§^ | 95%CI | P (interaction) |
| --- | --- | --- | --- | --- | --- | --- | --- | --- |
| ***Overall*** | | | | 3650 | 60.9 | - | 54.0, 68.8 | - |
| **Age (per 10 year increase)** | | | | 3650 | - | 0.99 | 0.92, 1.07 | - |
| **Sex** | | | |  |  |  |  | - |
|  | Male | | | 1355 | 50.1 | 1.0 (ref) |  | - |
|  | Female | | | 2295 | 66.9 | 1.3 | 1.0, 1.6 | - |
| ***Farming*** | | | |  |  |  |  | - |
|  | | None | | 1568 | 55.5 | 1.0 (ref) |  | - |
|  | | Part-time | | 668 | 54.9 | 1.0 | 0.7, 1.4 | - |
|  | | Full time | | 1414 | 69.0 | 1.1 | 0.9, 1.4 | - |
| **Daily hours spent in fields (per 1 hour increase)** | | | | 3650 | - | 1.01 | 0.99, 1.04 |  |
| **Distance from house to village edge (per 10m increase)** | | | | 3650 | - | 0.98 | 0.93, 1.03 | - |
| **Effect of sex by sero-prevalence within 200m** | | | |  |  |  |  | 0.463 |
|  | | | < 25% | 902 |  | 0.9 | 0.6, 1.6 |  |
|  | | | ≥ 25%, < 50% | 1415 |  | 1.6 | 1.1, 2.3 |  |
|  | | | ≥ 50% | 1333 |  | 1.4 | 0.9, 2.1 |  |
| **Effect of farming by sero-prevalence within 200m** | | | |  |  |  |  | 0.013 |
|  | | | < 25% | 902 |  | 2.2 | 1.2, 3.9 |  |
|  | | | ≥ 25%, < 50% | 1415 |  | 1.1 | 0.8, 1.6 |  |
|  | | | ≥ 50% | 1333 |  | 0.9 | 0.6, 1.3 |  |

^ǂ^Serologically apparent infection defined as sero-conversion from optical density <1 to ≥1 in baseline seronegative participants, or as an optical density increase of ≥1 in baseline seropositive participants; ^§^Rate ratio calculated using conditional log-log models (age, sex, farming, Daily hours spent in fields), or spatial Poisson models (Distance from house to village edge, effects by sero-prevalence within 200m). All models included age, sex, years of education and location of water source and daily hours of field work in the rainy season, except models with farming as exposure.

**Table S6.** Sensitivity analysis using sero-prevalence instead of sero-incidence as outcome

|  | | | | N | Prevalence^ǂ^ | Adjusted PRR^§^ | 95%CI | P (interaction)* |
| --- | --- | --- | --- | --- | --- | --- | --- | --- |
| ***Overall*** | | | | 5251 | 34.7 | - | 33.4, 36.0 | - |
| **Age (per 10 year increase)** | | | | 5251 | - | 1.17 | 1.12, 1.22 | - |
| **Sex** | | | |  |  |  |  | - |
|  | Male | | | 2001 | 29.8 | 1.0 (ref) |  | - |
|  | Female | | | 3250 | 37.7 | 1.2 | 1.1, 1.4 | - |
| ***Farming*** | | | |  |  |  |  | - |
|  | | None | | 2415 | 31.1 | 1.0 (ref) |  | - |
|  | | Part-time | | 927 | 27.3 | 1.0 | 0.8, 1.2 | - |
|  | | Full time | | 1909 | 42.8 | 1.2 | 1.1, 1.4 | - |
| **Daily hours spent in fields (per 1 hour increase)** | | | | 5251 | - | 1.01 | 1.00, 1.02 |  |
| **Distance from house to village edge (per 10m increase)** | | | | 5251 |  | 1.00 | 0.98, 1.03 |  |
| **Effect of sex by sero-prevalence within 200m** | | | |  |  |  |  | 0.349 |
|  | | | < 25% | 1256 |  | 1.0 | 0.7, 1.4 |  |
|  | | | ≥ 25%, < 50% | 2044 |  | 1.4 | 1.2, 1.7 |  |
|  | | | ≥ 50% | 1951 |  | 1.1 | 1.0, 1.2 |  |
| **Effect of farming by sero-prevalence within 200m** | | | |  |  |  |  | 0.624 |
|  | | | < 25% | 1256 |  | 1.2 | 0.9, 1.5 |  |
|  | | | ≥ 25%, < 50% | 2044 |  | 1.1 | 1.0, 1.3 |  |
|  | | | ≥ 50% | 1951 |  | 1.2 | 1.1, 1.3 |  |

^ǂ^Optical density cut-off to define sero-positivity of 2.0; ^§^prevalence risk ratio (PRR) calculated using Poisson regression models with robust standard errors adjusted for clustering at household level (age, sex, farming, daily hours spent in fields), or spatial Poisson models (Distance from house to village edge, effects by sero-prevalence within 200m).


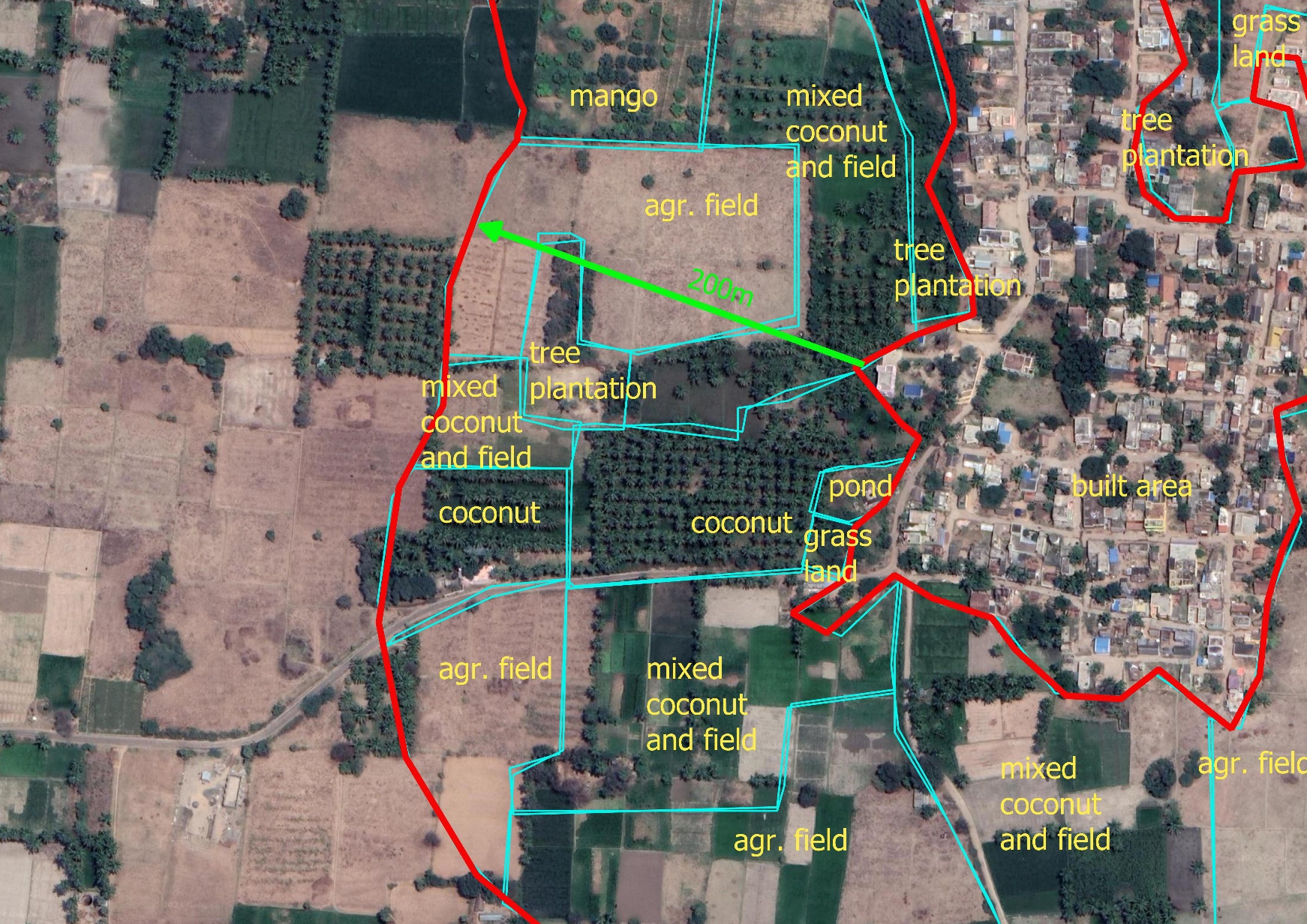


**Figure S1**. Manual land use classification. Red lines demark 200m buffer zone around the village.


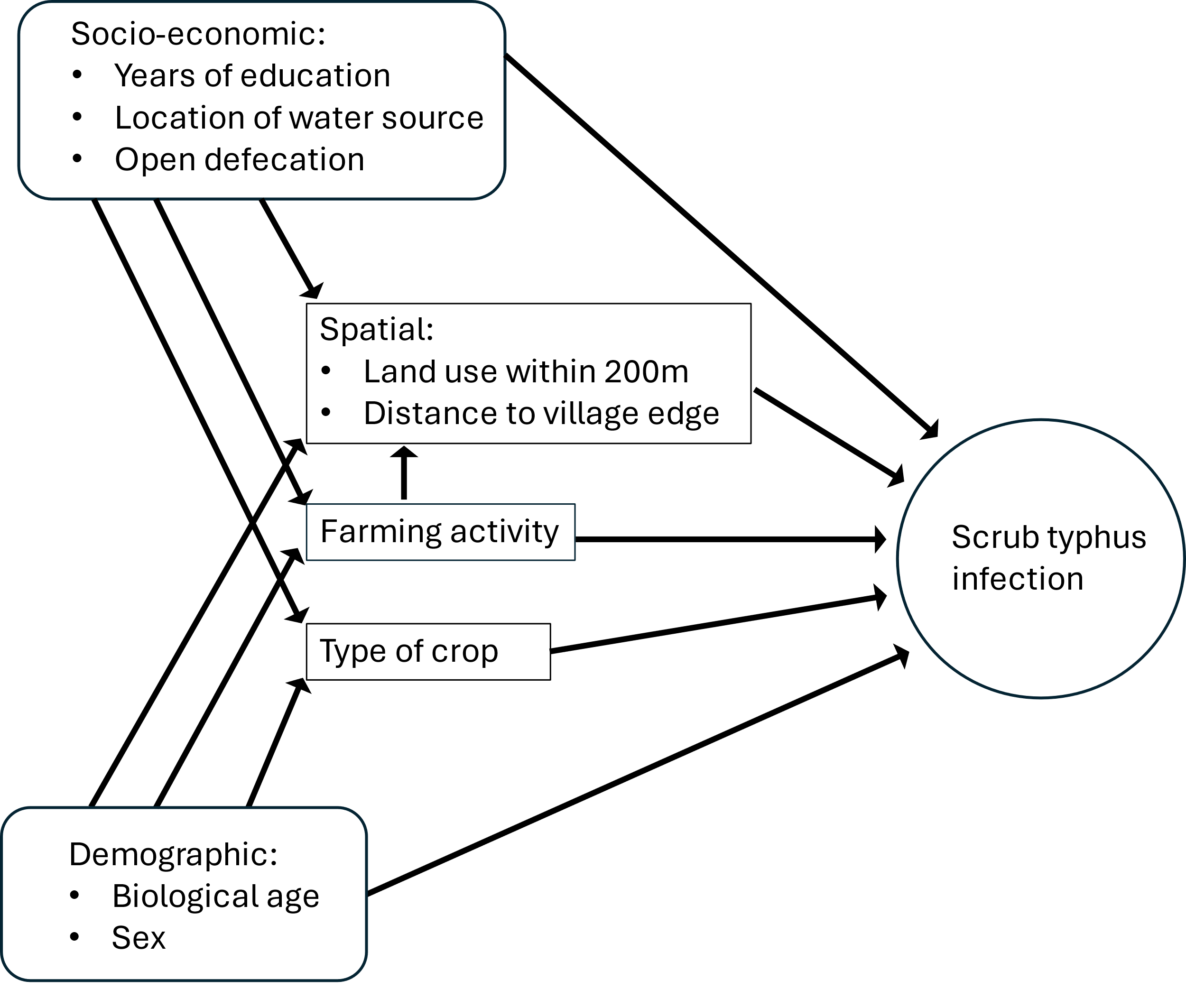


**Figure S2.** Directed acyclic graph of potential causal relationships between groups of exposure variables (Farming activities, type of crop, spatial variables), potential socio-economic and demographic confounders and scrub typhus infection
